# Is Scale-Up Worth It? Challenges in Economic Analysis of Diagnostic Tests for Tuberculosis

**DOI:** 10.1371/journal.pmed.1001063

**Published:** 2011-07-26

**Authors:** David W. Dowdy, Adithya Cattamanchi, Karen R. Steingart, Madhukar Pai

**Affiliations:** 1 Department of Medicine, University of California, San Francisco, California, United States of America; 2 Division of Pulmonary and Critical Care Medicine, San Francisco General Hospital, San Francisco, California, United States of America; 3 Curry International Tuberculosis Center, San Francisco, California, United States of America; 4 Department of Epidemiology, Biostatistics, and Occupational Health, McGill University, Montreal, Canada

## Abstract

David Dowdy and colleagues discuss the complexities of costing new TB diagnostic tests, including GeneXpert, and argue that flexible analytic tools are needed for decision-makers to adapt large-sample cost-effectiveness data to local conditions.

**Summary Points**

- Standard cost-effectiveness analyses may give misleading results when applied blindly to the scale-up of TB diagnostics.
- Challenges in economic analysis of TB diagnostic tests include: underestimating the cost of false-positive diagnoses, overlooking operational and clinical impact of diagnostics, and utilizing unrealistic cost-effectiveness thresholds.
- Solutions include: establishing society's valuation of false-positive tests, evaluating the consequences of TB misdiagnosis in field settings, and setting local cost-effectiveness thresholds for disease-specific interventions.
- Flexible and accessible analytic tools are needed for decision-makers to adapt large-sample cost-effectiveness data to local conditions.

## Background: Scaling Up Rapid Diagnostic Tests

Since 2007, the World Health Organization (WHO) has approved an unprecedented number of new diagnostic tests for tuberculosis (TB) [1],[2]. Most recently, Xpert MTB/RIF (Cepheid, Inc.; Sunnyvale, CA), an automated polymerase chain reaction (PCR) test with high accuracy in validation studies (72%–77% sensitivity for smear-negative TB, 99% specificity) [3],[4], was endorsed by WHO [5] and reduced in price [6]. To impact TB globally, Xpert MTB/RIF and other diagnostics must be scaled-up across numerous clinical settings, after careful evaluation of expected costs and benefits. Unfortunately, standard cost-effectiveness analyses are ill-suited to guide local decision-makers in directing scale-up activities. We demonstrate the limitations of standard economic analyses as applied to scale-up of TB diagnostics (specifically Xpert MTB/RIF), and recommend adaptations to future analyses that will facilitate rational and effective scale-up activities.

## Economic Analysis of TB Diagnostics: Current Practice

Decision analysis is the most widely-used methodology for evaluating health interventions' cost-effectiveness [7]. Decision analyses have assessed many TB diagnostics, including liquid culture [8], line probe assays [9], and theoretical point-of-care tests [10]. When applied to diagnostic tests, decision analysis must estimate the probability, economic cost, and effectiveness for each of four possible test results: true positive, true negative, false positive, and false negative. These quantities are calculated separately with and without a new diagnostic test; the incremental cost-effectiveness ratio (ICER) describes the difference in cost, divided by the difference in effectiveness, between the two scenarios. The ICER, often reported as the cost per disability-adjusted life year (DALY) averted, may be compared against a selected benchmark, such as per-capita gross domestic product (GDP) [11].

**Figure 1:**
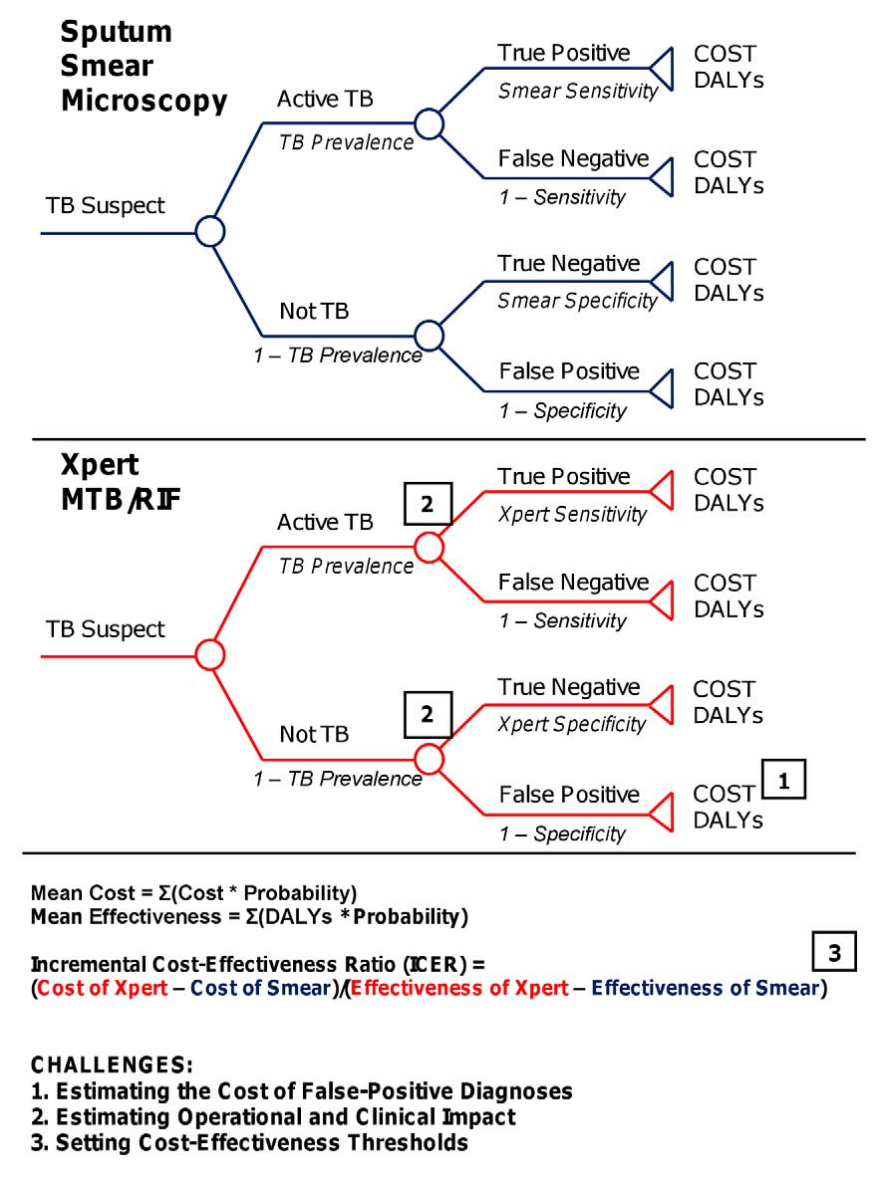
Schematic decision analysis. Decision tree for a hypothetical cost-effectiveness analysis comparing sputum smear microscopy (blue) against Xpert MTB/RIF (red). Circles represent chance nodes, where probabilities are applied to each branch as described in italics. Triangles represent terminal nodes, where costs and effectiveness are calculated. Squares demonstrate the points in the analysis at which the analytic challenges described in the text are encountered.

For example, a simple decision analysis might evaluate a hypothetical cohort of TB suspects undergoing diagnosis with sputum smear microscopy versus Xpert MTB/RIF (Figure 1). The number of true positives, true negatives, false positives, and false negatives (diagnostic outcomes) are calculated by applying test sensitivity and specificity to the cohort prevalence of active TB. Estimates from the literature or data from field evaluations inform the mean cost and effectiveness (in DALYs) for each of these four outcomes under the two diagnostic strategies. For each outcome, cost and effectiveness are multiplied by probability to estimate the overall cost and effectiveness of sputum smear versus Xpert MTB/RIF. Additional assumptions and calculations can expand the analysis to include other diagnostic tests or more faithfully represent the diagnostic process, but the probability, cost, and effectiveness of each outcome must be calculated to generate cost-effectiveness ratios. In these essential steps of decision analysis, three key challenges arise when evaluating TB diagnostics:

1. The costs of false-positive diagnoses are poorly defined and often underestimated.
2. Diagnostic accuracy (i.e., sensitivity and specificity) is an inadequate proxy of outcomes important to patients and public health.
3. Diagnostic testing often competes for resources with other TB-specific interventions, making standardized cost-effectiveness thresholds largely irrelevant.

## Challenge #1: Estimating the Cost of False-Positive Diagnoses

Whereas the costs of false-negative TB diagnoses can be summarized by projecting the consequences of untreated TB (including transmission), the costs of false-positive diagnoses are difficult to estimate. Published studies generally confine their estimates to the costs of diagnostic testing, inappropriate disease treatment, and management of medication side effects [12]. However, false-positive TB diagnoses may cause morbidity and mortality from other conditions for which treatment is delayed on the basis of a rapidly false-positive TB test. Furthermore, false-positive diagnosis may lead to overuse of TB drugs, increasing risks for acquired drug resistance. These costs to patients and society are not incorporated into most decision analyses, which therefore tend to overestimate the cost-effectiveness of TB diagnostics.

More importantly, the economic costs of TB treatment are miniscule relative to the costs of untreated TB. In fact, most analyses underestimate the costs of untreated TB by not accounting for the costs of transmission from untreated cases. Because untreated TB carries such high costs, standard analyses favor any diagnostic test that increases the number of TB cases treated, even if it generates more false-positive diagnoses than most physicians and patients would accept. For example, in Rwanda, it has been argued that treating 29 false-positives for every additional case of active TB would be cost-effective [13]. Similarly, a US$20 TB diagnostic test with 15% sensitivity and 50% specificity would be recommended on standard cost-effectiveness grounds [10]. However, it is unlikely that patients or physicians would accept a diagnosis that is wrong 29 times out of 30, or a test performing more poorly than a coin flip. Estimates of the true cost of false-positive TB diagnosis must account for these values and preferences.

The consequences of underestimating costs from false-positive diagnoses are magnified as diagnostic tests move from the laboratory to the field during scale-up. Even for diagnostics that demonstrate exceptional specificity in controlled settings (and for TB, where no existing test can prove absence of disease, specificity is difficult to determine), suboptimal performance is expected when used by health workers with little laboratory training or external quality control. In particular, molecular TB diagnostics have lower sensitivity and specificity when used outside the laboratory [14], due in part to higher rates of sample contamination [15]. Furthermore, TB prevalence is generally lower in field settings than in controlled studies, which appropriately enrich their populations with TB cases to maximize power. For example, Xpert MTB/RIF was initially tested in a population with 55% TB prevalence, demonstrating specificity of 99.2% and identifying 25 new smear-negative TB cases for each false-positive [3]. However, if implemented with 95% specificity in a field setting having 10% TB prevalence, where 50% of TB is smear-positive and 50% of smear-negative TB is detected clinically, Xpert MTB/RIF would identify 2.6 false-positives for every new smear-negative TB case. Thus, standard economic analyses of TB diagnostics relying on controlled studies to estimate sensitivity, specificity, and TB prevalence may simultaneously underestimate both the cost and frequency of false-positive TB diagnoses. Multiplying these figures to generate a cost-effectiveness ratio may result in considerable bias.

## Challenge #2: Estimating Operational and Clinical Impact

**Figure 2:**
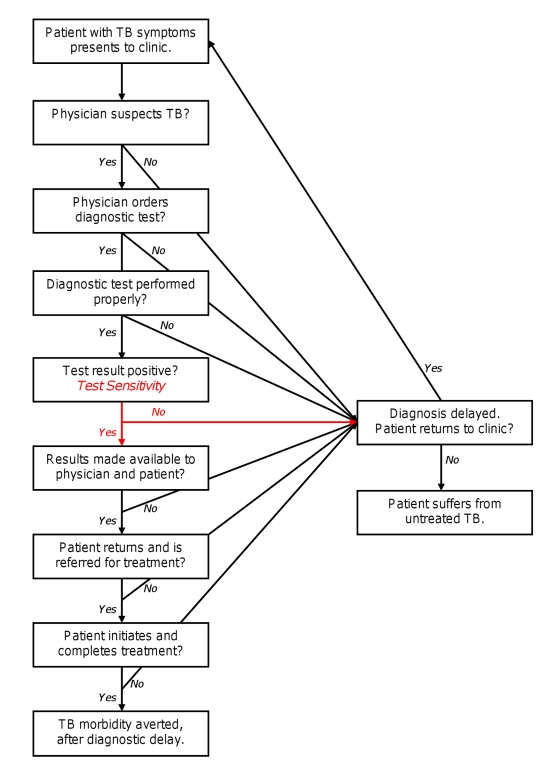
Process of TB diagnosis. Boxes represent steps in the diagnostic process that must be completed for patients to experience positive clinical outcomes. Accuracy of the diagnostic test (depicted in red) plays a necessary but small role.

Disease diagnosis and management is a complex and dynamic process, of which a test's diagnostic accuracy is only a small component (Figure 2). Throughout this process, patients' clinical manifestations progress, thresholds for empiric treatment evolve [16], and different members of the health care system interact. As a result, lab-based estimates of diagnostic accuracy may not correlate with operational or clinical impact in the field. For example, in one study of peripheral clinics in Uganda, only 21% of individuals with suspected TB were referred for microscopy, and 71% of patients with positive smears initiated TB treatment [17]. A typical analysis assuming that all individuals with suspected TB are tested and all true-positives are treated would greatly overestimate a diagnostic test's effectiveness under these conditions. Other operational realities rarely incorporated into analyses of TB diagnostics include empiric treatment (where diagnostic test results do not affect outcome), time delays in obtaining results, impact of test results on physician behaviors, difficulty in maintaining high-quality laboratory services, and disease progression with repeated testing (where initial false-negative results are subsequently corrected). Thus, the number of positive test results estimated from adding new diagnostics (e.g., Xpert MTB/RIF) to existing algorithms does not necessarily predict the number of positive clinical outcomes achieved. Operational data (e.g., [4]) must be incorporated as well.

## Challenge #3: Setting Cost-Effectiveness Thresholds

Public health resources in most countries are partitioned along disease-specific lines. Thus, scale-up of diagnostics often competes for resources against other interventions targeting the same disease. For TB, this might include additional infrastructure for directly observed therapy, or external quality assessment of microscopy. TB treatment is among the most cost-effective health interventions available. In Africa, for example, treating smear-positive TB costs US$8 per DALY averted [18]. Although there is no universal threshold for “cost-effectiveness,” many cost-effectiveness ratios are implicitly benchmarked against the annual per-capita GDP (≥US$300 in all countries except Zimbabwe [19]). Using this benchmark, a new TB diagnostic test costing US$170 per DALY averted [10] might appear economically favorable, but its scale-up could divert resources from other, more cost-effective interventions (such as expanded access to high-quality microscopy). Diversion of resources to scale-up rapid diagnostic tests is not simply a theoretical concern. In India, for example, providing Xpert MTB/RIF at current prices to 15% of all TB suspects would consume the entire annual budget of the Revised National TB Control Program (US$65 million in 2010) (D. Dowdy, K. Steingart, M. Pai, unpublished data).

## Improving Current Approaches

Scale-up of TB diagnostics will soon occur, with or without economic analyses to inform the process. Addressing the challenges outlined above will lead to better-informed policy recommendations and scale-up decisions, and ultimately to improved TB health outcomes worldwide. Many organizations, including the WHO, have adopted the Grading of Recommendations, Assessment, Development, and Evaluation (GRADE) approach to assessing quality of evidence and determining strength of recommendations for diagnostic tests and strategies [20]. An Impact Assessment Framework for TB diagnostics has also been proposed [21] in which scale-up analysis—including economic evaluation—informs policy analysis. The GRADE approach strongly considers patient-important outcomes, values and preferences, and resource use. Using these same principles to drive economic analyses of TB diagnostics will enhance policy relevance and provide more appropriate guidance to scale-up recommended diagnostic tests.

To estimate the cost of false-positive diagnoses, decision-makers should consider local preferences for decreasing false-positive versus false-negative test results. Simple surveys of patients, physicians, and members of society can be helpful. For example, clinicians in Ecuador, Laos, Nepal, and Rwanda were willing to treat two false-positives to prevent one undiagnosed case of TB [22]. For scale-up in this setting (from the physicians' perspective), an analysis should value the cost of false-positives as one-half that of false-negatives. When local preferences seem inappropriate to policy-makers, educational efforts or recommendations for empiric therapy should be prioritized over scale-up of novel diagnostics. Data should also be collected on the morbidity and mortality suffered by patients with other conditions who are inappropriately diagnosed and treated for TB.

To estimate the operational impact of rapid diagnostics, decision-makers need comparative data on patient- and provider-important outcomes in clinical sites with and without test access. Cluster-randomized trials (potentially with stepped-wedge randomization [23]) could provide such information. Study outcomes should include incidence and mortality (both disease-specific and all-cause), physician judgment (to estimate rates of empiric treatment), long-term follow-up (to characterize repeated diagnostic attempts), and quality-of-life surveys. Mathematical models could use these data to project the medium-term impact and cost-effectiveness of scaling-up TB diagnostics, ideally incorporating the “multiplier” effect of transmission. Before scaling-up new diagnostics, appropriate infrastructure must be developed to ensure that diagnostic results translate into patient outcomes [8].

To set appropriate cost-effectiveness thresholds, the activities that would be supplanted by scaling-up rapid diagnostics should be identified. Cost-effectiveness analyses could then better define the (willingness-to-pay) threshold at which new diagnostics should be scaled-up.

Ultimately, decisions regarding scale-up of rapid diagnostics will be made at the national or sub-national level, and relevant data will vary widely between locations and constituencies (e.g., public versus private sector). To be most effective, such decisions must take into account not only test accuracy and cost, but also the socioeconomic factors that drive most TB epidemics [24]. Model studies conducted in representative populations can inform broad guidelines, but local adaptation should be emphasized. This process may be facilitated by developing flexible and accessible analytic tools that combine data from larger studies with smaller evaluations of local preferences, practices, and economic conditions. At least one crude but prototypical tool based on a published analysis of hypothetical TB diagnostic tests [10] is currently available online [25].

## Conclusions

Standard cost-effectiveness analyses may give misleading results when applied blindly to the scale-up of TB diagnostics. To be useful to both policy-makers and decision-makers, such analyses should (1) establish society's valuation of false-positive tests relative to false-negative tests, (2) evaluate the consequences of false-negative and false-positive diagnoses when new diagnostics are deployed in field settings, and (3) set local cost-effectiveness thresholds for disease-specific interventions. Model studies and analytic tools allowing flexible user-defined inputs can help local decision-makers adapt broad policy guidelines to local conditions. Confronting these challenges will help ensure that innovations in TB diagnostic testing lead to improved patient and population health worldwide.

## Abbreviations

DALY: disability-adjusted life year
GDP: gross domestic product
GRADE: Grading of Recommendations, Assessment, Development, and Evaluation
TB: tuberculosis
WHO: World Health Organization
